# Preparation of Protein Molecular-Imprinted Polysiloxane Membrane Using Calcium Alginate Film as Matrix and Its Application for Cell Culture

**DOI:** 10.3390/polym10020170

**Published:** 2018-02-10

**Authors:** Dong Liu, Kongyin Zhao, Meng Qi, Shuwen Li, Guoqing Xu, Junfu Wei, Xiaoling He

**Affiliations:** 1 State Key Laboratory of Separation Membranes and Membrane Processes, Tianjin Polytechnic University, Tianjin 300387, China; techiec@163.com (D.L.); jfwei@tjpu.edu.cn (J.W.); 2 School of Material Science and Engineering, Tianjin Polytechnic University, Tianjin 300387, China; m18222610665@163.com (M.Q.); 1611530310@stu.tjpu.edu.cn (S.L.); coach_xu@yeah.net (G.X.); hexiaoling@tjpu.edu.cn (X.H.)

**Keywords:** calcium alginate, polysiloxane, molecular-imprinted polymer, bovine serum albumin adsorption, fibronectin, cell culture

## Abstract

Bovine serum albumin (BSA) molecular-imprinted polysiloxane (MIP) membrane was prepared by sol-gel technology, using silanes as the functional monomers, BSA as the template and CaAlg hydrogel film as the matrix. The stress-strain curves of wet CaAlg membrane and molecular-imprinted polysiloxane membrane were investigated. We evaluate the adsorption and recognition properties of MIP membrane. Results showed that the adsorption capacity of BSA-imprinted polysiloxane for BSA reached 28.83 mg/g, which was 2.18 times the non-imprinted polysiloxane (NIP) membrane. The adsorption rate was higher than that of the protein-imprinted hydrogel. BSA-imprinted polysiloxane membrane could identify the protein template from competitive proteins such as bovine hemoglobin, ovalbumin and bovine γ-globulin. In order to obtain the biomaterial that can promote cell adhesion and proliferation, fibronectin (FN)-imprinted polysiloxane (FN-MIP) membrane was obtained by using fibronectin as the template, silanes as functional monomers, and CaAlg hydrogel membrane as the substrate or matrix. The FN-MIP adsorbed more FN than NIP. The FN-imprinted polysiloxane membrane was applied to culture mouse fibroblast cells (L929) and the results proved that the FN-MIP had a better effect on cell adhesion than NIP.

## 1. Introduction

Biomaterial scaffold mimics the extracellular matrix and serves as a temporary skeleton for cell growth [1], migration, proliferation, differentiation and finally maturation [2,3,4]. Biomaterials that identify specific molecular potential have attracted a lot of attention to allow adsorption and adhesion of proteins that properly accommodate and rebuild the target tissue [5]. The studies on protein adsorption, protein recognition as well as the interaction between proteins and biomaterials have been widely studied [6,7,8]. 

Molecular imprinting technique is an effective method for the establishment of specific identification sites for target molecules in synthetic materials [9,10,11,12,13]. The obtained molecularly-imprinted polymers have complementary binding sites and template-specific recognition cavities, which can serve as repositories of drug molecules and improve drug residence time [14,15,16,17]. Molecular imprinting technology has been widely used in various small molecules and made an excellent progress. However, the imprinting of protein is still a major challenge owing to the complexity of surface structures, the high flexibility of conformation and diversity of protein sequences [18,19]. 

One of the interesting biomaterials is polyacrylamide, which is always used to prepare cross-linking hydrogels existing protein recognition sites [20,21,22,23]. Although polyacrylamide hydrogels have high selectivity to template proteins, they are short of ample thermo stability and mechanical strength. In addition, the diffusion of protein in the hydrogel is quite slow. Surface imprinting technique can be applied to solve the problem of protein diffusion [24,25,26]. As functional monomers, silanes and its derivatives were employed to synthetize molecular-imprinted polysiloxane on the surface of solid substrate. Organic silane is easy to polymerize by sol-gel process to create polysiloxane, which has high and low temperature resistance, hydrophobicity and physiological inertia. Most silanes contain functional groups that react with proteins. Mosbach et al. discussed the preparation of protein molecular imprinting polymers by organic silane monomers [27]. There were multiple hydrogen bonds between the silanol groups of polysiloxane and surface polar residues of proteins. The results show that the silicon dioxide coated with polysiloxane has affinity for the glycoprotein transferrin. Toru Shiomi et al. [28] synthesized protein molecular-imprinted polymer on silica using 3-aminopropyltrimethoxysilane and trimethoxypropylsilane as the functional monomers for the determination of hemoglobin (Hb). Two kinds of organic silanes were polymerized on a surface of porous silica after the Hb template was covalently immobilized by forming imine bonds. A sensitive silica gel microsphere capture protein was prepared by Kyoko Fukazawa et al. [29]. It was used for high selective identification of bovine serum albumin (BSA). The imprinted protein was immobilized to silica beads by using a phospholipid polymer containing both active ester groups and silane coupling groups. Zian Lin et al. [30] fabricated a macro porous silica monolith for protein molecular imprinting. In our previous work, BSA molecular-imprinted polysiloxane (MIP) was prepared with silanes as functional monomers, BSA as templates and calcium silicate containing mesoporous SiO_2_ on the surface (CaSiO_3_@SiO_2_) as the matrix [31]. However, the above-prepared MIP particles are inconvenient in some applications, especially in cell culture.

As one of the biological materials extracted from brown algae, calcium alginate has been successfully used in food, drug delivery, tissue engineering and beverages because of its biocompatibility [32]. Alginate-based hydrogels have been widely used for cell microencapsulation [33]. BSA-imprinted calcium alginate membrane has been prepared for the release of BSA from the alginate matrix [34]. Our previous work was to prepare BSA-imprinted polymers based on calcium alginate hydrogel microspheres [35]. However, the protein-imprinted microspheres are inconvenient in cell culture, especially when a cell sheet is required.

In this paper, calcium alginate (CaAlg) hydrogel membranes were synthesized by cross-linking sodium alginate (SA) in CaCl_2_ solution. BSA molecular-imprinted polysiloxane polymer was prepared using β-methoxyethylene triethyoxysilane (KH-570) and γ-amidopropyl triethyoxysilane (KH-550) as functional monomers, BSA as template and CaAlg hydrogel membrane as the matrix. The molecular-imprinted polysiloxane (MIP) membrane was analyzed by means of transmission electron microscope (TEM), scanning electronic microscopy (SEM), and Fourier transform infrared spectroscopy (FT-IR). The adsorption and recognition properties of MIP membrane were evaluated. In order to obtain the biomaterial that can promote cell adhesion and proliferation, fibronectin (FN)-imprinted polysiloxane was prepared by using FN as template, silanes as functional monomers, and CaAlg hydrogel membrane as the matrix. The results of cell culture showed that the FN-imprinted polysiloxane had a better effect on cell adhesion than NIP. 

## 2. Materials and Methods 

### 2.1. Materials

Sodium alginate (SA, chemical grade, *M*_W_ = 3.1 × 10^5^) was bought from Tianjin Northern China Medical Chemical Reagent (Tianjin, China). Oxalic acid and calcium chloride (CaCl_2_, analytical grade) was obtained from Shanghai Shen Xiang Chemical Reagent Co., Ltd. (Shanghai, China). Tianjin Shengbin Chemical Plant (Tianjin, China) supplied β-methoxyethylene triethyoxysilane (KH-570) and γ-amidopropyl triethyoxysilane (KH-550). Bovine serum albumin (BSA, *M*_W_ 67 kDa, pI 4.9), ovalbumin (Ova, *M*_W_ 43 kDa, pI 4.7), bovine hemoglobin (Hb, *M*_W_ 64.0 kDa, pI 6.9) and bovine γ-globulin (Glo, *M*_W_ 160kDa, pI 7.1) were bought from Lanji of Shanghai Science and Technology Development Company (Shanghai, China). The Cellular Biology Institute of the Chinese Academy of Sciences (Shanghai, China) supplied mouse fibroblast cells (L929). Fibronectin (FN, 440KD) was bought from Qianchen Biological Technology Company (Shanghai, China). MTT Cell Proliferation Cytotoxicity Assay Kit was from Wuhan doctor DE bioengineering Co., LTD. 

### 2.2. Apparatus

Ultraviolet spectrophotometer (TU-1901) was offered by Beijing Puxi general instrument Co., Ltd., Beijing, China. The full temperature oscillation incubator (HZQ-F) was from Donglian electronic technology development Co., Ltd., Harbin, China. Electronic balance (FA2004N) was achieved from Shanghai precision science instrument Co., Ltd., Shanghai, China. Field emission electron microscope (S-4800) was from Hitachi Co., Ltd., Tokyo, Japan. Constant temperature magnetic stirrer (85-2) was supplied by Shanghai Sile instrument factory, Shanghai, China. The infrared spectrometer (Nicolet iS50) was form Thermo Scientific Corp. New York, NY, USA. Integrated thermal analyzer (SDTQ600) was achieved from TA Instruments-Waters Ltd., Shanghai, China. Single fiber tensile testing device (LLY-06F) was supplied by Laizhou electronic instrument Co., Ltd., Laizhou, China. 

### 2.3. Preparation of Calcium Alginate (CaAlg) Hydrogel Membranes

The calcium alginate (CaAlg) hydrogel membranes were prepared according to the literature [36,37]. Firstly, different amounts of SA powders (0.3046, 0.4082, 0.5128, 0.6186 and 0.7254 g) were added individually into five beakers that contained 20 mL water and the mixture was stirred for 2 h. Then SA solutions with the SA content of 1.5%, 2.0%, 2.5%, 3.0% and 3.5% were obtained respectively. We put aside the SA solutions for 12 h to eliminate bubbles after ultrasonic processing for 5 min. Then we scraped 3–10 g of the viscous SA solution into membranes on a glass plate using a glass rod twined copper wire with the diameter of 0.4 mm. The glass plate with the SA solution was put into CaCl_2_ solutions with the concentration of 1.5%, 2.5%, 3.5% and 5.0% respectively for cross-linking 5 h. The resulting CaAlg hydrogel membranes were stored in CaCl_2_ aqueous solution (1%) for future use.

### 2.4. Preparation of CaAlg Hydrogel Based MIP and NIP Membranes

Approximately 0.100 g CaAlg membrane was placed in vials and 10 mL of 1.34 mg/mL BSA solution was added. After 12 h 0.050 mL KH-570 and 0.050 mL KH-550 silanes were added into the vials. The vials were incubated at 20 °C for 48 h for the hydrolysis and condensation polymerization of two silanes. Then the supernatant was removed and the CaAlg membrane coated with polysiloxane were washed with 10 mL oxalic acid solution (0.5 mol/L) to remove the template BSA. After washing the membrane with distilled water for removal of oxalic acid, BSA molecular-imprinted polysiloxane membrane was prepared and was noted as MIP (BSA-MIP). 

Simultaneously, non-imprinted polysiloxane was also fabricated according to the above procedures and named as NIP when the BSA solution was replaced with distilled water. 

Fibronectin (FN)-imprinted polysiloxane membrane was prepared by using FN as the template according to the above method, and the resulting imprinted polysiloxane was named as FN-MIP.

### 2.5. Characterizations

The CaAlg membranes prepared with 3.0 wt % SA, 2.5 wt % CaCl_2_ and the MIP membranes by using the CaAlg membranes as the matrix were used for characterization. We measured the thicknesses of CaAlg and MIP membranes in wet form with a digital display micrometer gauge provided by Shanghai Luchuan Co., LTD.

We examined the morphologies of CaAlg and MIP membranes by using a scanning electron microscope (SEM). Transmission electron microscopy (TEM) was used to investigate the microscopic morphology of MIP membrane at 200 kV accelerating voltage. Epoxy resin embedding and ultrathin sectioning were used to prepare TEM samples on a Leica Ultracut UCT ultramicrotomer. 

We investigated the chemical structures of CaAlg and MIP membranes by Fourier transform infrared spectrometry (FT-IR).

We tested the mechanical properties of the CaAlg and MIP membranes in wet form by using a tensile testing machine (LLY-06F).

### 2.6. Adsorption of BSA on BSA-MIP and NIP Membranes

About 100 mg MIP and NIP membranes were put into the glass bottle containing 0–2.00 mg/mL BSA aqueous solution to determine the adsorption isotherms, respectively. On order to determine the adsorption dynamics and the imprinting efficiency (*IE*) of MIP and NIP membranes, 1.36 mg/mL BSA aqueous solution was used. An ultraviolet spectrophotometer was used to measure the concentration of the above BSA aqueous solution at certain time intervals. According to Equation (1), the equilibrium adsorption capacity (*Q_e_*) (mg/g) of protein on the MIP and NIP membrane was determined.

*Q_e_* = (*C_0_* − *C_e_*) *V*/*W* (1)

where *V* (mL) is the volume of BSA solution, *C_0_* and *C_e_* (mg/mL) is the initial and final BSA concentration (mg/mL), and *W* (g) is the mass of dried MIP or NIP membrane. 

The imprinting efficiency (*IE*) of MIP membrane was defined as follows:

*IE* = *Q_MIP_/Q_NIP_* (2)

where *Q_MIP_* and *Q_NIP_* are the adsorption capacity of MIP membrane and NIP membrane, respectively.

### 2.7. Recognition Performance of BSA-MIP Membrane

The MIP and NIP membranes were put into each glass bottle, which contains 10 mL 1.36 mg/mL Hb, Ova, Glo and BSA solutions to assess the recognition performances of MIP [26].

### 2.8. Adsorption of FN on FN-MIP and NIP Membrane

About 20 mg of FN-MIP or NIP membrane was placed in a glass bottle containing 1 mL 0.10 mg/mL FN solution at room temperature. Then the Bradford method was used for protein quantitation at specific time intervals [38]. The adsorption capacity (mg/g) and the imprinting efficiency (*IE*) of FN-MIP membrane was calculated according to Section 2.6. 

### 2.9. Cell Culture

We sterilized the fibronectin (FN)-imprinted polysiloxane (FN-MIP) and NIP membranes by 70% (*v*/*v*) ethanol and washed them with sterile water. The FN-MIP and NIP membranes were placed into a glass bottle containing 5 mL 0.2 mg/mL FN aqueous solution for adsorption 2 h. Then the FN adsorbed FN-MIP and NIP membranes were placed into a culture plate (96-well). We seeded L929 cells on the FN-MIP and NIP membranes at a density of 2 × 10^4^ cells/well. The culture plate was cultured under a humidified atmosphere containing 5% CO_2_ at 37 °C. The morphologies of L929 cells cultured on NIP and FN-MIP membranes were examined by a microscope and the adhesion behavior of the cells was investigated according to the literature [39]. 

The growth of cells on the membranes was checked by the MTT assay. The culture medium was replaced with 1 mL 0.5 mg/mL fresh culture medium containing MTT. The excess medium was removed and 500 μL DMSO was added in order to dissolve the formazan crystal after incubating at 37 °C for 4 h. Then 150–200 μL DMSO solutions were poured into 96-well plates and the OD values of the each well were determined at 492 nm by a micro plate reader.

## 3. Results and Discussion

### 3.1. Characterizations of MIP Membranes

#### 3.1.1. Morphologies of MIP Membranes

**Figure 1:**
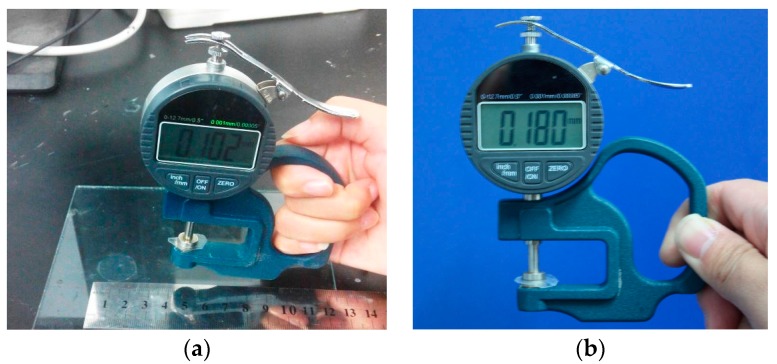
Digital photos of CaAlg membrane (**a**) and MIP membrane (**b**).

**Figure 2:**
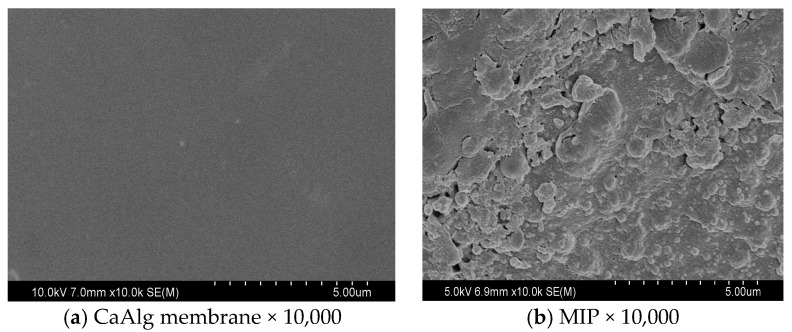
SEM images of CaAlg membrane (**a**) and MIP membrane (**b**).

**Figure 3:**
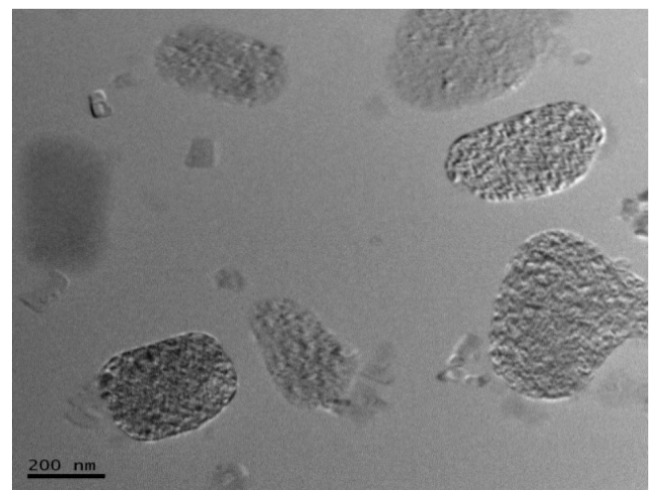
TEM images of MIP membrane.

The digital images of CaAlg and MIP membranes in wet form are showed in Figure 1. The transparency of CaAlg membrane was higher than MIP membrane. The polysiloxane on the surface of MIP film increased the thickness of the membrane (from 0.102 ± 0.019 to 0.180 ± 0.028 mm) and decreased the transparency of CaAlg membrane. Surface SEM images display the CaAlg membrane (Figure 2a) was smoother than that of MIP membrane (Figure 2b). The roughness of NIP membrane was similar to that of MIP membrane because they were both coated with the polysiloxane. Figure 3 is the TEM image of MIP membrane, in which many BSA-imprinted polysiloxane particles with a diameter of around 200–320 nm were identified.

#### 3.1.2. Identification of the Membrane Formation 

**Figure 4:**
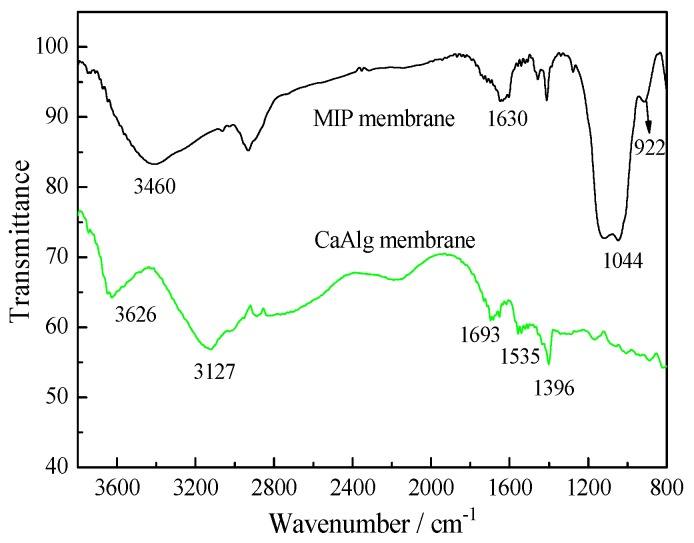
FT-IR spectra of CaAlg membrane and MIP membrane.

Figure 4 shows the FT-IR spectra of CaAlg membrane and MIP membrane. The –OH group of the CaAlg exhibited the peak at 3626 cm^−1^. The strong absorption of –COO^−^ in CaAlg was observed at 1610~1500 and 1500~1400 cm^−1^. The strong absorption peak of –COO^−^ from CaAlg was weakened in the MIP membrane, indicating the form of cross-linked structure between CaAlg and the polysiloxane. The MIP membranes showed strong adsorption at 922 and 1044 cm^−1^, which was attributed to the Si–O–Si stretching vibration. The significant difference between CaAlg and MIP membranes appeared at 922 cm^−1^, revealing that the polysiloxane of MIP membrane was created on the surface of CaAlg membrane.

#### 3.1.3. Mechanical Properties of CaAlg and MIP Membrane

**Figure 5:**
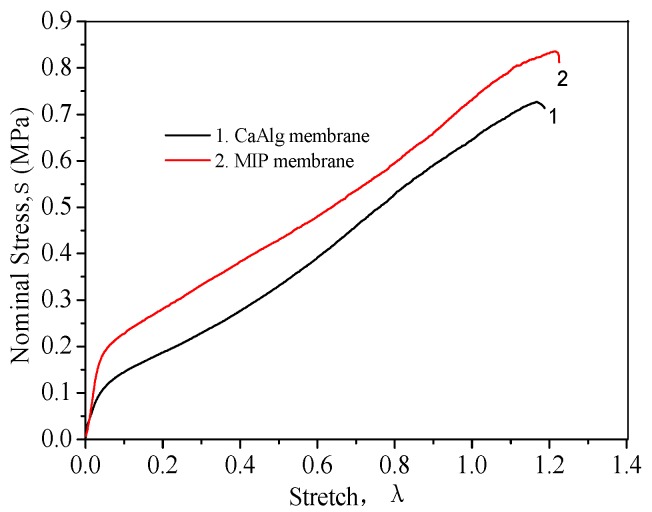
Stress–strain curves of CaAlg membrane and MIP membrane.

Figure 5 shows the stress-strain curves of CaAlg membrane and the MIP membrane in wet form. The MIP membrane was stronger than CaAlg membrane because the interactions between the polysiloxane and alginate. The silane coupling agent KH-550 molecules have been diffused to alginate and adsorb on CaAlg shells through electrostatic interaction between the protonated amino groups in KH-550 and the carboxylate groups in alginate [40]. The condensation of KH-570 and KH-550 occurred to come into being a polysiloxane network on the CaAlg membrane surface. So the MIP membranes showed increased mechanical properties.

### 3.2. Adsorption of MIP Membrane Prepared with Different CaCl_2_ and SA Concentrations

**Figure 6:**
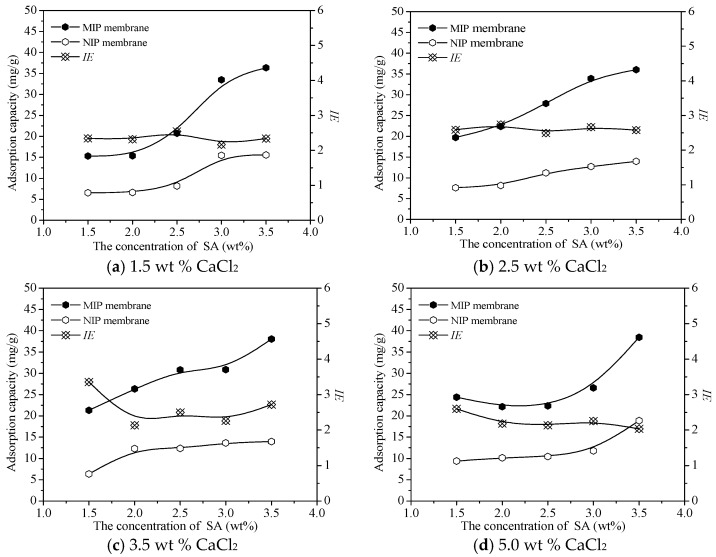
Adsorption capacity and *IE* of the MIP membrane prepared with different CaCl_2_ and SA concentrations.

Figure 6 shows the adsorption capacity and the *IE* of MIP membrane prepared with different CaCl_2_ and SA contents. The adsorption capacity of MIP and NIP membrane increased with the raise of the SA concentration because of the formation of more polysiloxane on the surface of CaAlg membrane prepared with a higher SA content. The concentration of CaCl_2_ didn’t work on the adsorption quantity of MIP and NIP membranes. The MIP membrane adsorbed more protein than the NIP membrane did and the *IE* reached over 2.0. However, the influence of the concentration of SA and CaCl_2_ on the *IE* was not significant. Based on the mechanical performance of CaAlg membranes, 2.5 wt % CaCl_2_ and 3.0 wt % SA were used for the preparation of CaAlg and the MIP membranes in the following studies. 

### 3.3. Adsorption Kinetics of BSA on MIP and NIP Membrane

**Figure 7:**
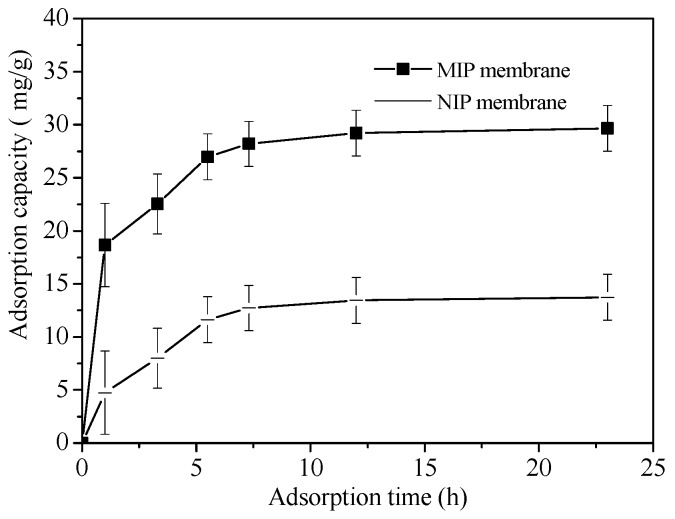
Adsorption dynamics of BSA on MIP and NIP membrane.

Figure 7 shows the BSA adsorption dynamics curves on MIP and NIP membrane. Within 5.5 h, the adsorption rate was high and the equilibrium adsorption capacity (*Q_e_*) reached 75% for both NIP and MIP membrane. The adsorption rate of the BSA-imprinted polysiloxane in this paper was much faster than that of the BSA-imprinted hydrogel, which took 24 h to reach the equilibrium adsorption [37]. After 5.5 h the *Q_e_* of MIP membrane was 2.18 times higher than NIP membrane, with a value of 28.83 mg/g. The adsorption capacity of BSA by NIP membrane at 5.5 h was 13.52 mg/g. The higher adsorption capacity of BSA on MIP was due to the form of the reacted sites and the complementary cavities between BSA and the MIP membrane. Because the sites and the imprinting cavities were on the surface of the MIP membrane and BSA could spread more easily, so the MIP membrane had a faster adsorption rate for its template. 

### 3.4. Adsorption Thermodynamics of BSA on MIP and NIP Membrane

**Figure 8:**
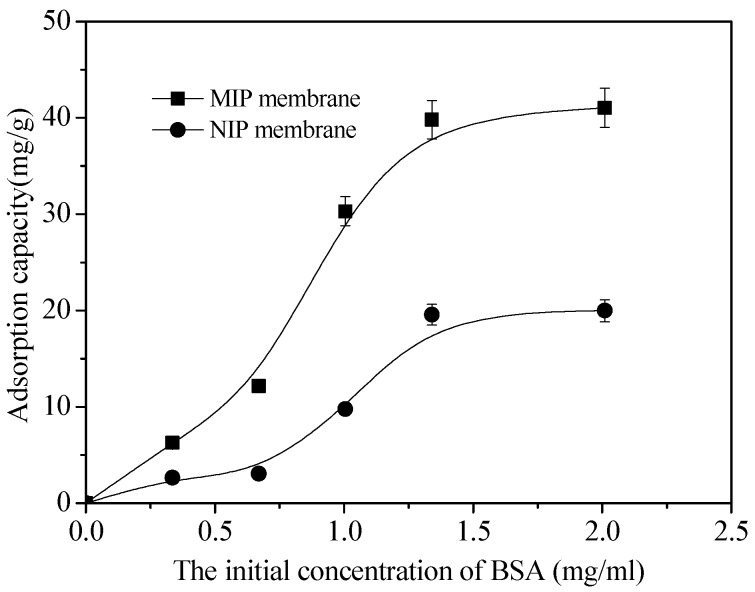
Curves of adsorption thermodynamics of MIP and NIP membrane.

Figure 8 shows the adsorption thermodynamics of BSA on MIP and NIP membrane. With the increase of initial BSA concentration, the adsorption capacity increased rapidly and gradually tended to balance. The equilibrium adsorption capacity (*Q_e_*) of BSA on MIP membrane was more than 2.05 times than that of NIP membrane. When the concentration of BSA is low, the amount of BSA is not sufficient to fill the specific binding hole and cavity, so the *Q_e_* increases with the increase of BSA concentration. The adsorption capacity of MIP film tends to be stable when almost all the imprinting sites are occupied. 

**Figure 9:**
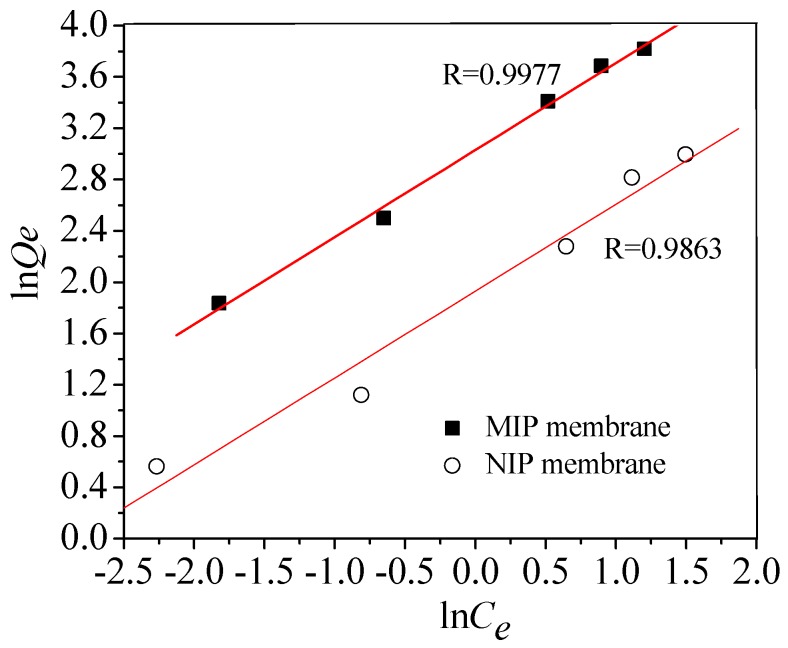
Curves of ln*Q_e_* to ln*C_e_* of MIP and NIP membrane.

The rebinding curves of NIP and MIP membrane can be followed by the Freundlich model equation:

*Q_e_* = *Q_f_* · *C_e_*^1/*n*^ (3)

where *Q_f_* is the rough rebinding capacity (mg/g), *C_e_* is the equilibrium concentration of BSA (mg/mL), and 1/*n* is the adsorption intensity. The linearized plots of ln*Q_e_* versus ln*C_e_* for the NIP and MIP membrane are showed in Figure 9. The ln*Q_e_* versus ln*C_e_* all exhibited good linearity with an *R* > 0.98.

### 3.5. Specific Adsorption of BSA on MIP and NIP Membrane

**Figure 10:**
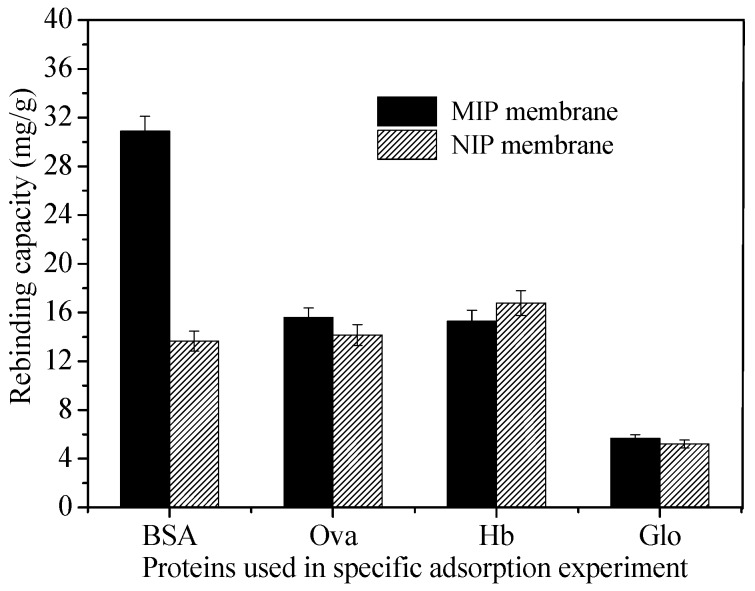
Adsorption capacity of MIP membrane and NIP membrane for BSA, Ova, Hb and Glo, respectively.

Figure 10 shows the *Q_e_* of MIP and NIP membrane for BSA and the competitive protein Ova, Hb and Glo, respectively. It is demonstrated that the BSA-imprinted membrane showed good adsorption selectivity for the template BSA. The *Q_e_* of BSA on the MIP membrane was 2.2 times as much as that of Hb or Ova, and 5.4 times as much as that of Glo. The higher BSA adsorption capacity of the MIP membrane is due to the generation of BSA affinity sites and the complementary cavities in the polycondensation of functional silanes. The recognition sites of BSA were not complementary to the Hb, Ova and Glo. So the competitive proteins (Ova, Hb and Glo) were less likely to be adsorbed on the BSA-imprinted membrane. In contrast, the NIP membrane adsorbed much less BSA than MIP membrane because there were no specific recognition sites on NIP membrane in the absence of template proteins. The molecular volume of Glo was larger than that of BSA and the other competitive proteins, so the *Q_e_* of γ-Glo on MIP membrane was much lower than that of any other proteins. 

### 3.6. Rebinding Behavior of FN on FN-MIP and NIP Membrane

**Figure 11:**
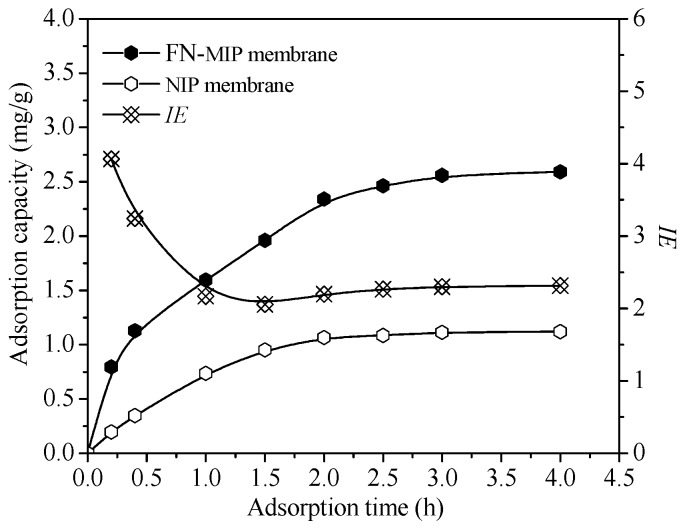
The adsorption dynamic curves of FN on FN-MIP and NIP membrane.

Fibronectin (FN) can promote cell growth, increase cell wall rate and enhance cell metabolism. If a material can selectively bind FN, then it has better biocompatibility. However, FN is very expensive. FN molecularly imprinted polymers (FN-MIP) was prepared according to the optimum condition of BSA-imprinted polymer. The adsorption dynamic curves of FN on FN-MIP and NIP membrane are shown in Figure 11. It is found that the adsorption capacity almost reached equilibrium after 3.0 h. The FN-MIP adsorbed more FN than NIP, and the *IE* reached 2.3. The adsorption capacity of FN was much lower than that of BSA, because its initial concentration is only one tenth of BSA. The molecular weight of FN is 440 kD, which is significantly greater than BSA (6.8 kD). In the rebinding process, the hole in the material is more difficult to allow FN in and out. The adsorption happened mainly on the surface. So the time of adsorption equilibrium was shorter than that of BSA.

### 3.7. Cell Culture on FN-MIP and NIP Membranes

**Figure 12:**
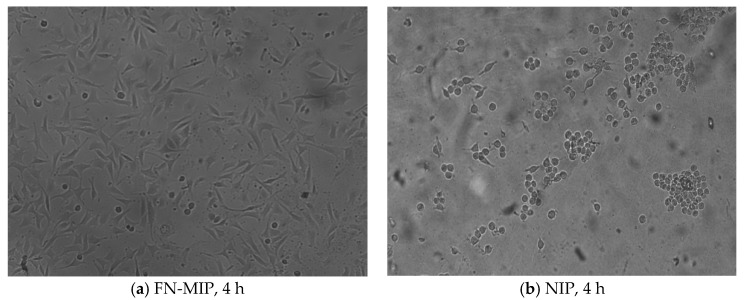
Photographs of adhesive L929 cells on MIP and NIP after cultured for 4 h.

Fukazawa et al. [41] provided a new molecular imprinting method using fibronectin (FN) to capture cells as a template. In this paper, FN-imprinted polysiloxane membrane was prepared according to the optimum conditions of BSA molecular imprinting. After adsorption of FN, L929 cells were cultured on the FN-MIP and NIP membrane, and Figure 12 shows the morphologies of L929 cells on the FN-MIP and NIP membranes after 4 h. The amount of cells adhered to FN-MIP membrane was much more than the cells adhered to the NIP membrane, indicating that FN-MIP membrane was more appropriate for culturing cell than NIP membrane. 

**Figure 13:**
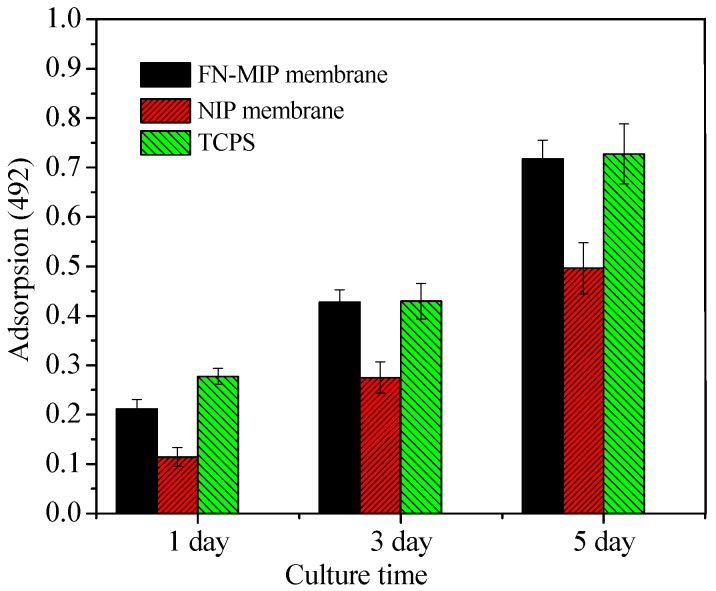
MTT assay of L929 cell-viability on FN-MIP membrane, NIP membrane, and TCPS (*p < 0.05).

Figure 13 shows cell-viability cultured with FN-MIP membrane, NIP membrane and tissue culture polystyrene board (TCPS) after 1, 3 and 5 days in culture, respectively. L929 cells on FN-MIP membrane grew more rapidly than those on NIP membrane during 5 days of incubation. Statistical analysis showed that the live cells on FN-MIP membrane were higher than those on NIP membrane because the FN-MIP membrane could adsorb more FN than NIP membrane, which made it was more suitable for the adhesion and growth of cells. Pan et al. [42,43] introduced the cell-adhesive peptide (RGDS) onto a thermo-responsive cell culture substrate. The substrate could be used as a highly efficient novel system. The FN-imprinted polysiloxane based on CaAlg membrane is low cost and easy to be prepared, which would show its great potential applications in cell sheet technology. 

## 4. Conclusions

Bovine serum albumin (BSA) molecular-imprinted polysiloxane (MIP) membrane was successfully synthesized using β-methoxyethylene triethyoxysilane (KH-570) and γ-amidopropyl triethyoxysilane (KH-550) as functional monomers, BSA as template and CaAlg hydrogel film as the matrix. The strength of MIP membrane was higher than that of CaAlg membrane. The stable adsorption capacity of BSA on MIP membrane was 2.18 times higher than that of non-imprinted polysiloxane (NIP) membrane, with a value of 28.83 mg/g. BSA-imprinted polysiloxane membrane could recognize BSA by using bovine hemoglobin (Hb), ovalbumin (Ova) and bovine γ-globulin (Glo) as competitive proteins. 

Fibronectin (FN)-imprinted polysiloxane membrane was prepared by using FN as the template according to the optimum conditions of BSA molecular imprinting. The FN-MIP adsorbed more FN than NIP, and the *IE* reached 2.3. After adsorption of FN, L929 cells were cultured on the FN-MIP and NIP membrane, and the results showed that the FN-imprinted polysiloxane (FN-MIP) exhibited better cell adhesion performance than the NIP. L929 cells on FN-MIP membrane grew more rapidly than those on NIP membrane.
